# Classification molecular subtypes of hepatocellular carcinoma based on PRMT-related genes

**DOI:** 10.3389/fphar.2023.1145408

**Published:** 2023-02-22

**Authors:** Liwen Liu, Qiuyue Hu, Yize Zhang, Xiangyi Sun, Ranran Sun, Zhigang Ren

**Affiliations:** ^1^ Precision Medicine Center, The First Affiliated Hospital of Zhengzhou University, Zhengzhou, China; ^2^ Department of Pharmacy, The First Affiliated Hospital of Zhengzhou University, Zhengzhou, China

**Keywords:** PRMT, hepatocellular carcinoma, molecular subtypes, prognostic molecular tool, immunotherapy, antineoplastic drug

## Abstract

**Background:** Recent studies highlighted the functional role of protein arginine methyltransferases (PRMTs) catalyzing the methylation of protein arginine in malignant progression of various tumors. Stratification the subtypes of hepatocellular carcinoma (HCC) is fundamental for exploring effective treatment strategies. Here, we aim to conduct a comprehensive analysis of PRMTs with bioinformatic tools to identify novel biomarkers for HCC subtypes classification and prognosis prediction, which may be potential ideal targets for therapeutic intervention.

**Methods:** The expression profiling of PRMTs in HCC tissues was evaluated based on the data of TCGA-LIHC cohort, and further validated in HCC TMA cohort and HCC cell lines. HCC was systematically classified based on PRMT family related genes. Subsequently, the differentially expressed genes (DEGs) between molecular subtypes were identified, and prognostic risk model were constructed using least absolute shrinkage and selection operator (LASSO) and Cox regression analysis to evaluate the prognosis, gene mutation, clinical features, immunophenotype, immunotherapeutic effect and antineoplastic drug sensitivity of HCC.

**Results:** PRMTs expression was markedly altered both in HCC tissues and HCC cell lines. Three molecular subtypes with distinct immunophenotype were generated. 11 PRMT-related genes were enrolled to establish prognostic model, which presented with high accuracy in predicting the prognosis of two risk groups in the training, validation, and immunotherapy cohort, respectively. Additionally, the two risk groups showed significant difference in immunotherapeutic efficacy. Further, the sensitivity of 72 anticancer drugs was identified using prognostic risk model.

**Conclusion:** In summary, our findings stratified HCC into three subtypes based on the PRMT-related genes. The prognostic model established in this work provide novel insights into the exploration of related therapeutic approaches in treating HCC.

## Introduction

Liver cancer is the third leading cause of death resulting from cancer worldwide, and it is responsible for 8.3% of all cancer deaths and poses a great health challenge (Sung et al., 2021; Vogel et al., 2022). Hepatocellular carcinoma (HCC) is a major histologic subtype of liver neoplasms (Tumen et al., 2022). Recent years, considerable attention has been paid to exploring novel systemic treatments for HCC patients, especially immune checkpoint inhibitors (ICIs) (Rizzo et al., 2022; Viscardi et al., 2022). Clinical response to ICIs including atezolizumab, nivolumab, and pembrolizumab have been evaluated in HCC patients (Rizzo et al., 2021). However, despite the numerous available treatment strategies, survival rate for HCC patients remains low due to postoperative recurrence, early distant metastasis, and dismal immunotherapeutic response (Lin et al., 2017; Forner et al., 2018). Considering its rising prevalence and worse clinical outcomes, more predictive biomarkers and therapeutic strategies for HCC deserve to be explored.

Protein arginine methylation, catalyzed by protein arginine methyltransferases (PRMTs), is emerging as a critical posttranslational modification (PTM) in various cell biological processes, such as epigenetic regulation, DNA damage response and immune surveillance (Jarrold and Davies, 2019; Dai et al., 2022). Generally, PRMT family enzymes serve as “writers” of PTM, are classified into three types according to the specificity of product: type I PRMTs (PRMT1, PRMT2, PRMT3, PRMT4, PRMT6, and PRMT8) mainly generate asymmetric arginine demethylation (aDMA); type II PRMTs including PRMT5 and PRMT9 that catalyze the formation of symmetric arginine dimethylation (sDMA). PRMT7, identified as type III PRMTs, catalyzes MMA only (Al-Hamashi et al., 2020; Wang et al., 2021; Dai et al., 2022). Recently, aberrant methylation of arginine residues has been implicated in several malignancies and the ectopic expression of PRMTs has been demonstrated in multiple types of tumors including HCC (Elakoum et al., 2014; Hernandez et al., 2017; Zhong et al., 2018; Song et al., 2020; Lei et al., 2022). Further, genetic or pharmacological targeting of PRMTs enhances the anti-cancer effects (Wang et al., 2019; Huang et al., 2021; Janisiak et al., 2021). To gain a holistic insight into the mechanism of PRMTs and successfully develop the effective inhibitors for HCC precise treatment, comprehensive analysis of PRMTs in HCC is urgently needed.

In this work, we first analyzed the expression patterns of PRMTs and screened PRMT-related genes to classify molecular subtypes of HCC. The molecular tools were constructed by analyzing the differences among three subtypes and the functional roles of our prognostic risk model in HCC were further determined. In summary, our findings provide strong evidence for PRMTs to be valuable biomarkers and therapeutic targets in HCC.

## Results

### Expression and mutation status of PRMTs in HCC

**FIGURE 1:**
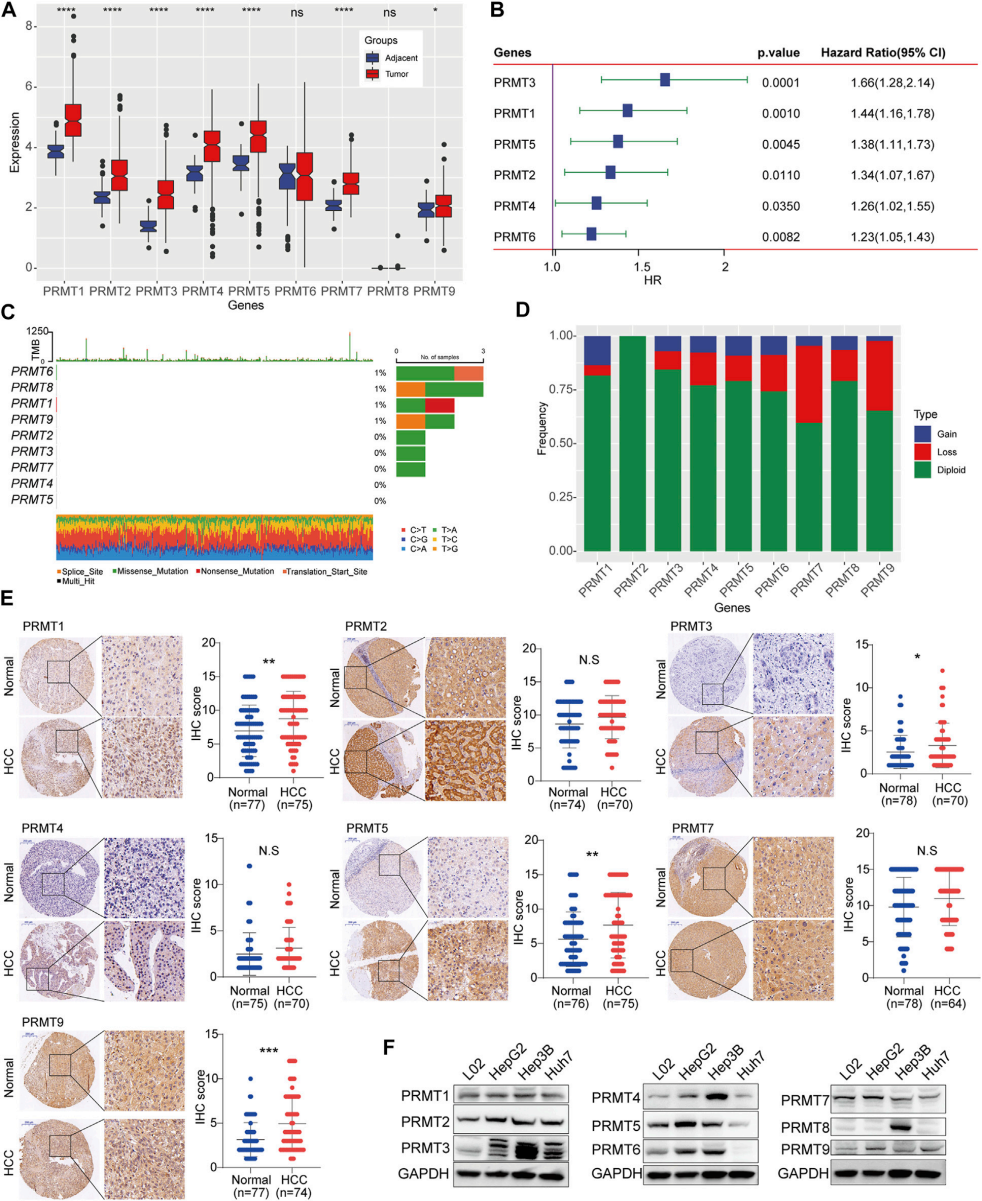
Expression and mutation status of PRMTs in HCC **(A)** The expression profiling of 9 PRMTs between HCC tissues and adjacent normal tissues. **(B)** PRMTs significantly associated with the prognosis of HCC were displayed in the forest map. **(C)** The waterfall plot represents somatic mutation status of 9 PRMTs in HCC. **(D)** The histogram exhibits the CNV pattern of each PRMT family genes. **(E)** Representative images of IHC staining of PRMTs in HCC TMA cohorts (left). IHC score of 7 proteins in HCC tissues and adjacent normal tissues were further quantified (right). **(F)** The protein level of 9 PRMTs in HCC cell lines and normal liver cell was determined by western blot.

Firstly, we identified the expression patterns of PRMTs in HCC based on TCGA-LIHC dataset. Among the 9 PRMTs, the expressions level of PRMT1, PRMT2, PRMT3, PRMT4, PRMT5 and PRMT7 and PRMT9 in HCC tissues were frequently overexpressed than those in adjacent normal tissues (Figure 1A). Further, PRMT1, PRMT2, PRMT3, PRMT4 and PRMT5 and PRMT6 were significantly correlated with patient’s poor survival (Figure 1B). Somatic mutations were also found in PRMT1, PRMT6, PRMT8, and PRMT9 genes (Figure 1C). In terms of copy number variation (CNV), there was no CNV in PRMT2, and the main CNV in PRMT1 was copy number gain, while the main CNV mode in PRMT3, PRMT4, PRMT5, PRMT6, PRMT 7, PRMT8 and PRMT9 was copy number loss (Figure 1D). Then the protein expression status of PRMTs in HCC tissues was identified based on HCC TMA cohort. Results showed that PRMT1, PRMT3, PRMT5, and PRMT9 were remarkedly upregulated in HCC tissues compared with matched non-neoplastic counterparts (Figure 1E). Further, we evaluated the expression level of 9 PRMT proteins in several HCC cell lines and an obviously altered expression status of PRMTs in HCC cell lines were observed (Figure 1F). These data suggest PRMTs were distinctly differentially expressed in HCC.

### Three molecular subtypes of HCC were stratified according to PRMT related genes

**FIGURE 2:**
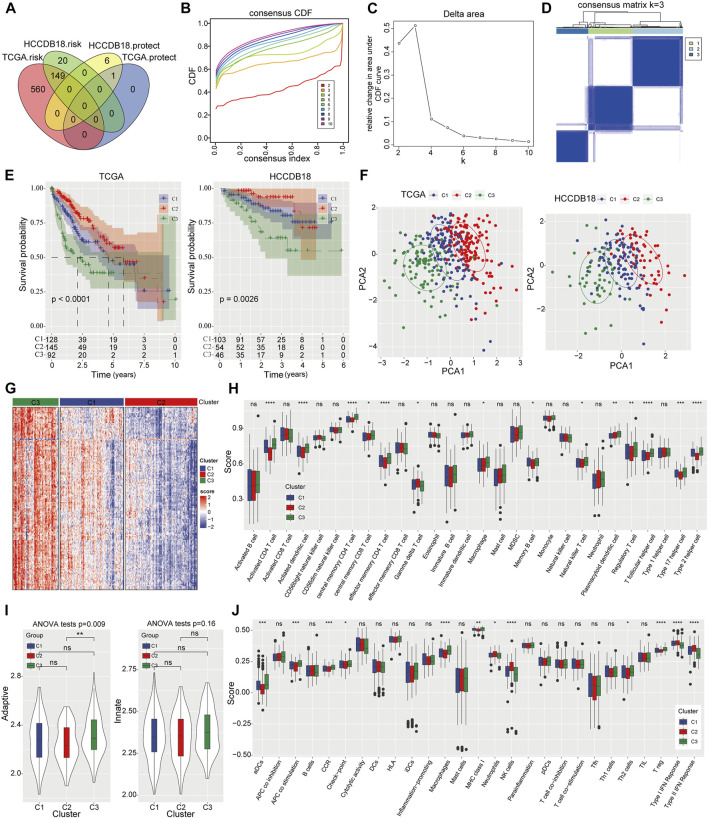
Identification of three PRMT related subtypes of HCC and immune status evaluation **(A)** The Venn diagram shows the PRMT-related genes with potential prognostic value that coincide between the TCGA-LIHC project and the HCCDB18 dataset. **(B, C)** The CDF curve trend and Delta area with k at 2–10. **(D)** Cluster heat map for consensus matrix with k = 3. **(E)** The Kaplan-Meier analysis among three molecular subtypes in TCGA-LIHC cohort and HCCDB18 cohort. **(F)** The distribution of consensus clusters by PCA. **(G)** Heat map represents enrichment score distribution of immune genes in three HCC subtypes. **(H)** The content of 28 immune cells quantified by ssGSEA in three HCC subtypes. **(I)** The difference of adaptive immunity and innate immunity among the three subsets. **(J)** The enrichment degree of 29 immune signatures in three clusters.

A total of 1,452 genes were identified as PRMT-related genes through correlation analysis. And 710 genes associated with prognosis in TCGA-LIHC dataset and 176 prognosis-related genes in HCCDB18 dataset were selected out *via* Univariate Cox regression analysis, respectively. After overlapping the above genes in two projects, 149 risk genes and 1 protect gene were finally filtered out for HCC stratification (Figure 2A). Subsequently, the CDF curve trend and the inflection point of Delta area curve were analyzed, and we choose 3 as the cluster classification number (Figures 2B, C). Thereafter, three molecular subtypes of HCC were obtained (Figure 2D). Compared to C1 and C3 molecular subtypes, patients in C2 subtype had significantly better prognosis in TCGA-LIHC dataset and HCCDB18 dataset respectively (Figure 2E). Significant difference in subgroups distribution and obvious aggregation in each group were observed through PCA analysis (Figure 2F). Meanwhile, the enrichment scores of immune-related genes among three subtypes were prominently different, with the highest enrichment score in C3 and the lowest enrichment score in C2 (Figure 2G). The abundance among three HCC subtypes were significantly different in immune cells quantified by ssGSEA (Figure 2H). The adaptive and innate immune activity among three subsets was also evaluated (Figure 2I). Further, the enrichment degree of 12 immune signatures among the subgroups were examined and we found that the enrichment levels of activated dendritic cells (aDCs), antigen presenting cells (APCs) co-stimulation, check-point, cytokine and cytokine receptor (CCR), macrophage, MHC class I, Th2 cells, regulatory T-cells (Treg) in C2 group were significantly downregulated. Whereas, neutrophils, NK cells and type I/II IFN response in C2 group were obviously upregulated (Figure 2J). Functionally, GSEA analysis of C2 subgroup with the best OS results and C3 subgroup with the worst OS results showed that retinol metabolism and fatty acid metabolism were remarkably enriched in this C2 subtype (Supplementary Figure S1A). Signaling pathways including pathways in cancer, DNA replication, cell cycle and homologous recombination were mainly enriched in C3 subgroup (Supplementary Figure S1B).

### Prognostic risk model construction and validation based on PRMT related genes

Next, we conducted differential expression analysis between HCC C1 and C3 subtypes, C1 and C2 subtypes, and C2 and C3 subtypes respectively (Supplementary Figures S2A–C). A total of 45 overlapped genes (PRMT; survival-related genes) were obtained among DEGs in three HCC molecular subtypes (Supplementary Figure S2D). The Univariate Cox regression identified 25 genes that were closely related to disease outcome of HCC patients (Supplementary Figure S3A) and then LASSO regression analysis was performed to penalize the linear model and reduce the complexity of the regression model (Supplementary Figures S3B, C). After simplifying the model using Multivariate Cox regression analysis, 11 PRMT-related genes were finally filtered out for prognostic risk model establishment: Risk score = 0.314 × ECT2 + 0.528 × KIF20A + 0.538 × CENPA-0.327 × TOP2A-0.805 × CCNB2-0.195 × PKM-0.28 × AURKB + 0.424×CDC20 + 0.131 × G6PD-0.059 × SLC22A1-0.069 × ADH4 (Supplementary Figure S3D).

**FIGURE 3:**
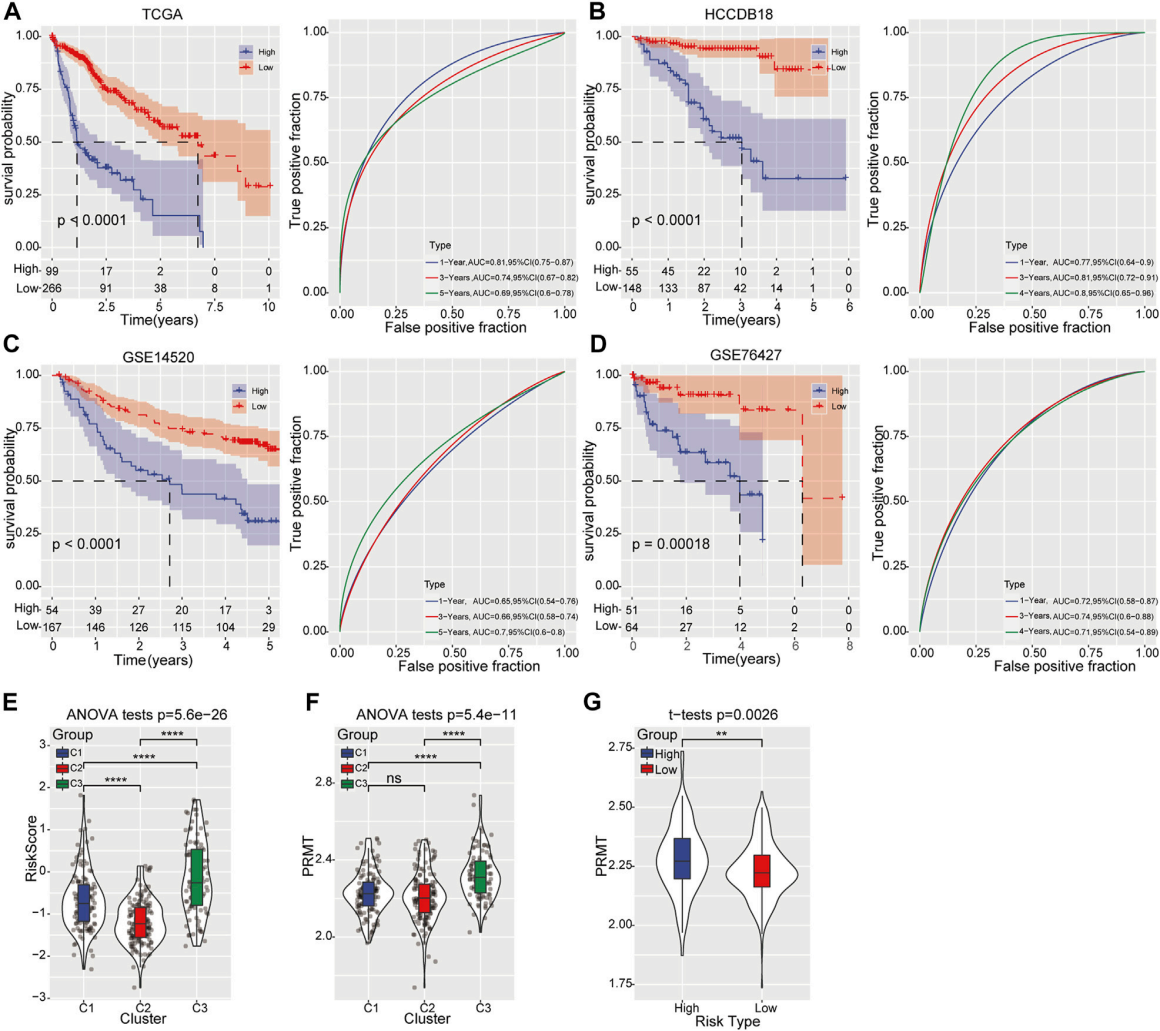
Prognostic risk model construction and validation **(A)** Kaplan-Meier curve (left) displays the OS of HCC patients predicted by prognostic risk model in TCGA-LIHC training cohort. The predictive performance was determined by ROC curve (right). **(B–D)** Kaplan-Meier curve (left) represents the OS of HCC patients validated in HCCDB18, GSE14520, and GSE76427 cohort respectively. The predictive ability was determined by ROC curve (right). **(E)** The distribution of risk score among the three molecular subtypes. **(F)** The expression of PRMTs among three molecular subtypes. **(G)** The level of PRMTs in the high- and low-risk groups.

To investigate the clinical significance of our risk model, HCC patients were divided into high- and low-risk groups based on the best cutoff value. Remarkable prognostic difference was observed between two groups and the clinical outcome of patients with high-risk score was obviously worse than those in low-risk categories. The area under the receiver operating characteristic (ROC) curve was 0.81 (95% CI 0.75–0.87) in predicting 1-year OS in training samples. For validating group, the maximum AUC was 0.81 (95% CI 0.72–0.91) for 3-year OS in HCCDB18 cohort, 0.7 (95% CI 0.60–0.80) for 5-year OS prediction in GSE14520, and 0.74 (95% CI 0.6–0.88) for 3-year OS in GSE76427 cohort (Figures 3A–D). Meanwhile, risk score displayed significant differences among three molecular subtypes: C2 with the best survival outcome had the lowest risk score, while C3 with the worst survival outcome had the highest risk score (Figure 3E). More importantly, the expression level of PRMTs in C3 subtypes was notably elevated than that in C1 and C2 (Figure 3F). Likewise, compared to low-risk category, the expression intensity of PRMTs in high-risk category was also significantly stronger (Figure 3G).

### Correlation analysis of prognostic risk model with mutation and clinicopathological features

**FIGURE 4:**
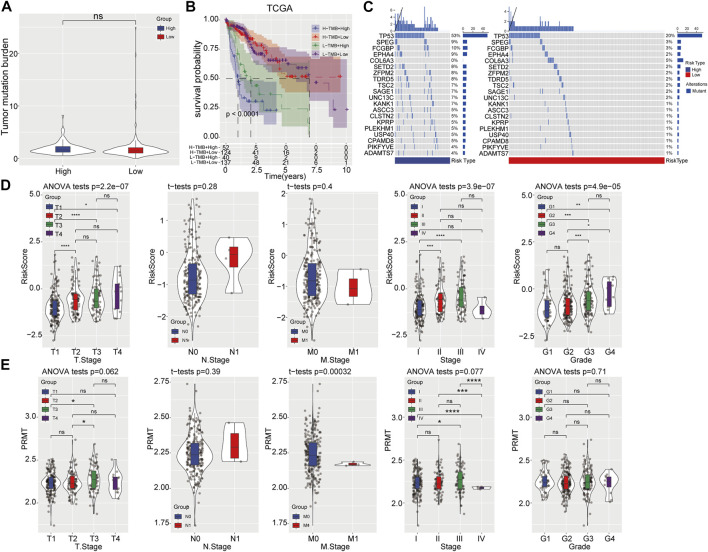
Correlation analysis of prognostic risk model with mutation and clinicopathological features. **(A)** TMB status in high- and low-risk categories. **(B)** The prognostic risk model combined with TMB to predict the prognosis of HCC. **(C)** Gene mutation rates in high- and low-risk categories. **(D, E)** Comparison of risk score and PRMT expression level among patients with different clinicopathological features.

Subsequently, the tumor mutation burden (TMB) distribution analysis was performed and no significant difference was found between two risk groups (Figure 4A). However, the combination of TMB and prognostic risk grouping system could significantly distinguish the clinical outcome (Figure 4B). Moreover, different mutation rates of genes were observed between the high-risk and low-risk categories, with the mutation rate of TP53 up to 53% in the high-risk category and 20% in the low-risk category. SPEG, FCGBP, EPHA4, SETD2 and other genes showed a higher mutation rate in the high-risk category than that in the low-risk category (Figure 4C). Further, the clinical value of the prognostic risk model was evaluated in TCGA-LIHC cohort and result showed that higher risk scores was positively associated with advanced clinical T stage, TNM stage and grade, but not M stage (Figure 4D). Moreover, high PRMT expression was also closely corelated with advanced TNM stage in HCC (Figure 4E).

### Effect of prognostic risk model on immune infiltration and drug response

**FIGURE 5:**
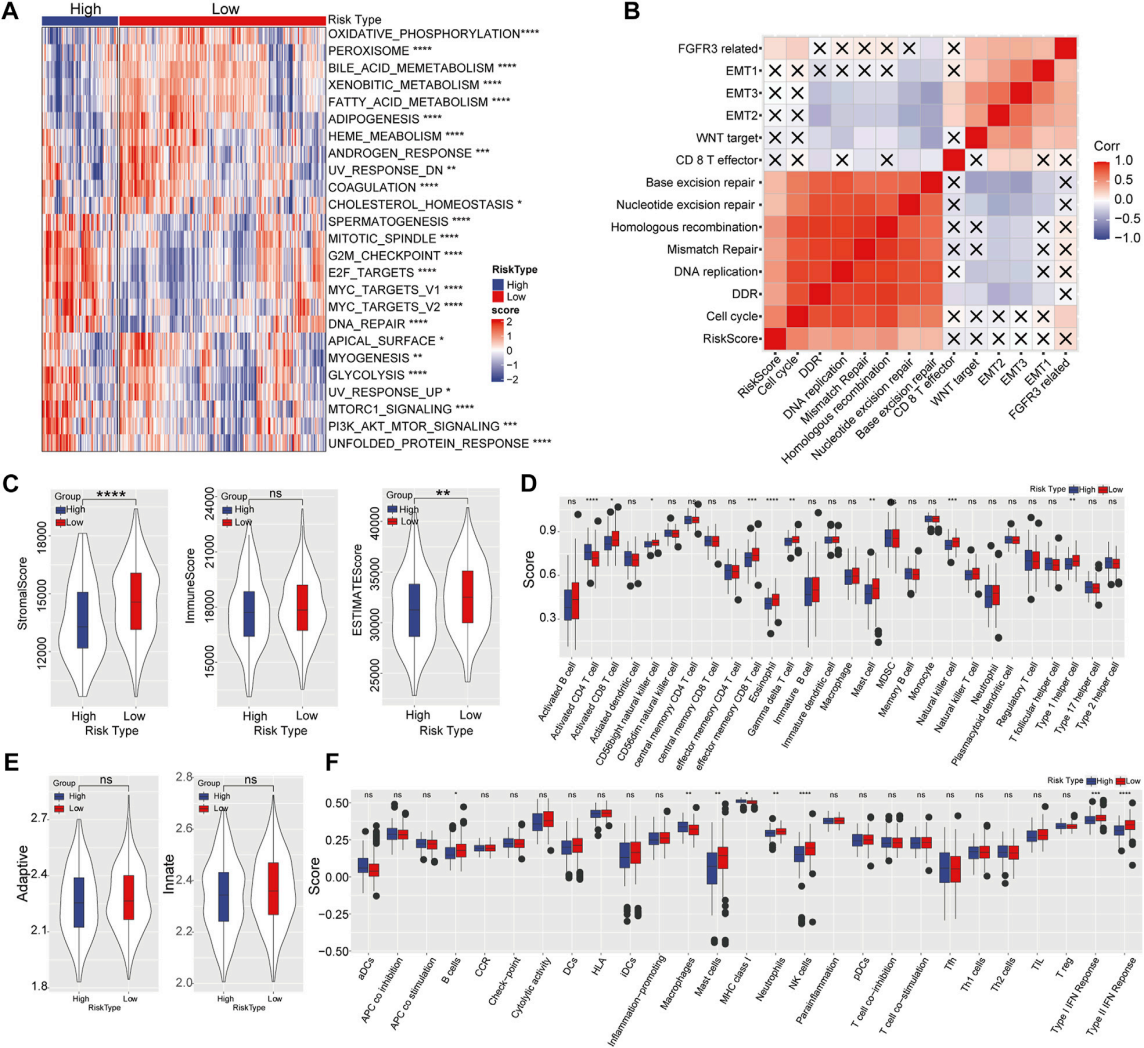
Effect of prognostic risk model on immune infiltration. **(A)** Enrichment heatmap of biological pathways in high- and low-risk categories. **(B)** Correlation analysis between risk score and biological process. **(C)** Stromal score, immune score, and ESTIMATE score in high-risk and low-risk categories. **(D)** The enrichment scores of 28 immune cells in high- and low-risk groups. **(E)** The adaptive and innate immune status in two risk groups. **(F)** The enrichment scores of 29 immune signature in high- and low-risk categories.

Then GSEA was performed to investigate biological pathways which closely related to highly risk in HCC patients. We found that majority pathways related to tumorigenesis were highly enriched in high-risk samples, including PI3K_AKT_MTOR SIGNALING, MTORC1_SIGNALING, E2F_TARGETS and G2M_CHECKPOINT (Figure 5A). Further correlation analysis showed a remarkable correlation between the risk score and cell cycle, as well as DNA damage repair (DDR), nucleotide excision repair and base excision repair (Figure 5B). HCC subgroup with high-risk score exhibiting lower stromal score and ESTIMATE score (Figure 5C). Meanwhile, ssGSEA revealed that the content of activated CD4^+^ T-cell in the high-risk category significantly increased, while the enrichment of activated CD8^+^ T-cell, effector memory CD8^+^ T-cell, gamma delta T-cell, eosinophil, CD56 bright natural killer cell, mast cell and type I T helper cell greatly decreased (Figure 5D). No significant difference was observed in adaptive and innate immune between high- and low-risk categories (Figure 5E). Additionally, higher levels of B cell, mast cell, neutrophils, NK cell, and type I/II IFN response were detected in the low-risk category, whereas macrophages and MHC class I at higher levels in the high-risk category (Figure 5F).

**FIGURE 6:**
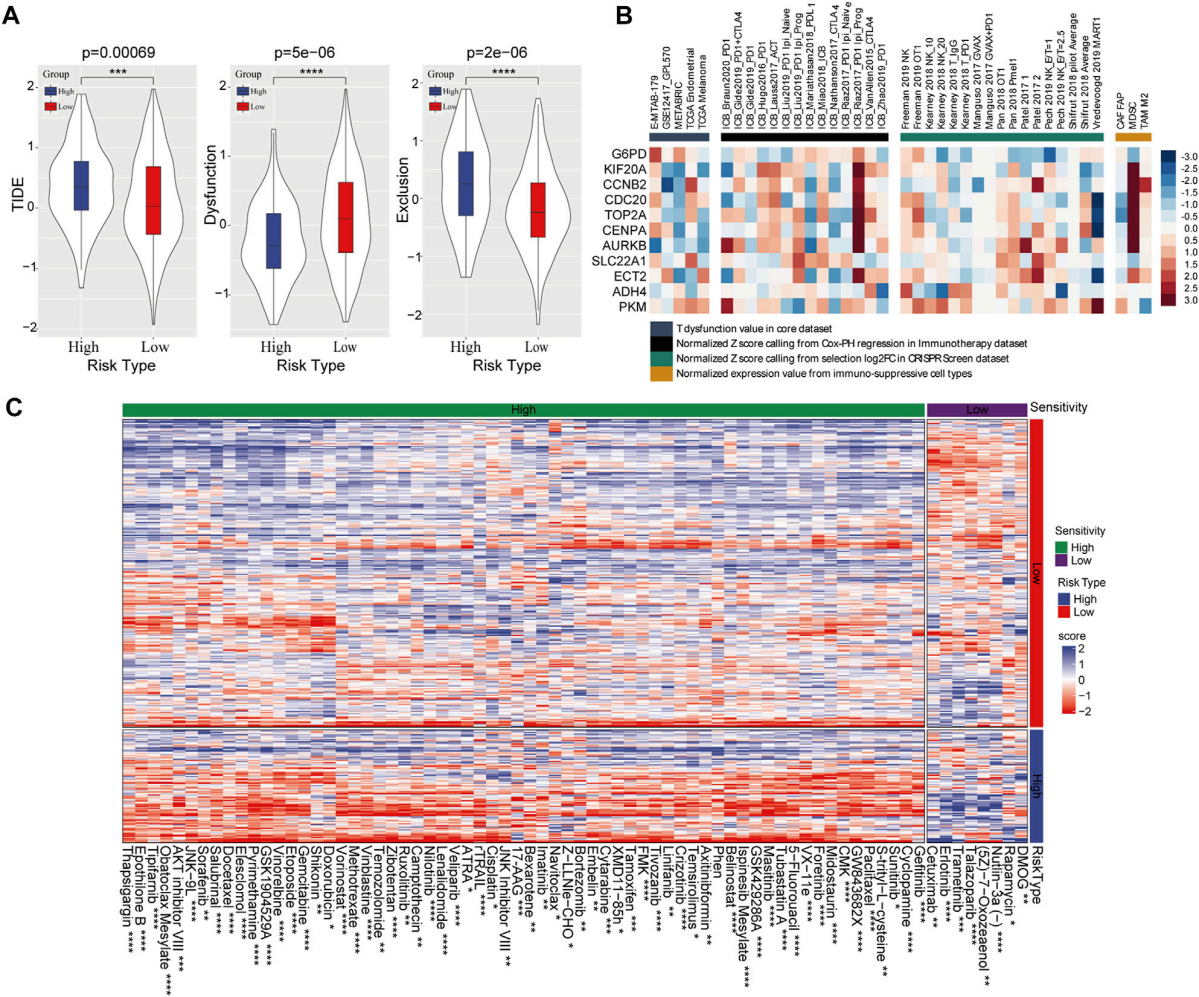
Effect of the prognostic risk model on immune response and drug sensitivity. **(A)** Comparison of TIDE score, T-cell dysfunction and T-cell exclusion scores in high- and low-risk group. **(B)** The correlation between 11 PRMT-related risk genes and T-cell dysfunction, ICB response and phenotypes in genetic screens and immune suppressive cell types. **(C)** Sensitivity of 72 chemotherapeutic drugs in high-risk and low-risk categories.

Subsequently, TIDE was used to predict risk score-based clinical immunotherapy responses. Significantly higher score of TIDE and T-cell exclusion, while lower score of T-cell dysfunction were found in high-risk categories, indicating that the high-risk category presented less sensitivity to immunotherapy (Figure 6A). Likewise, the 11 genes in prognostic risk model were associated with T-cell dysfunction, ICB response and immune-suppressive cell types (Figure 6B). Meanwhile, 72 chemotherapeutic or targeted drugs that showed differential sensitivity between high- and low-risk categories were determined using pRRophetic, of which 64 were highly sensitive in high-risk categories and 8 were highly sensitive in low-risk categories (Figure 6C).

### Evaluation the prognostic risk model on clinical immunotherapeutic response

**FIGURE 7:**
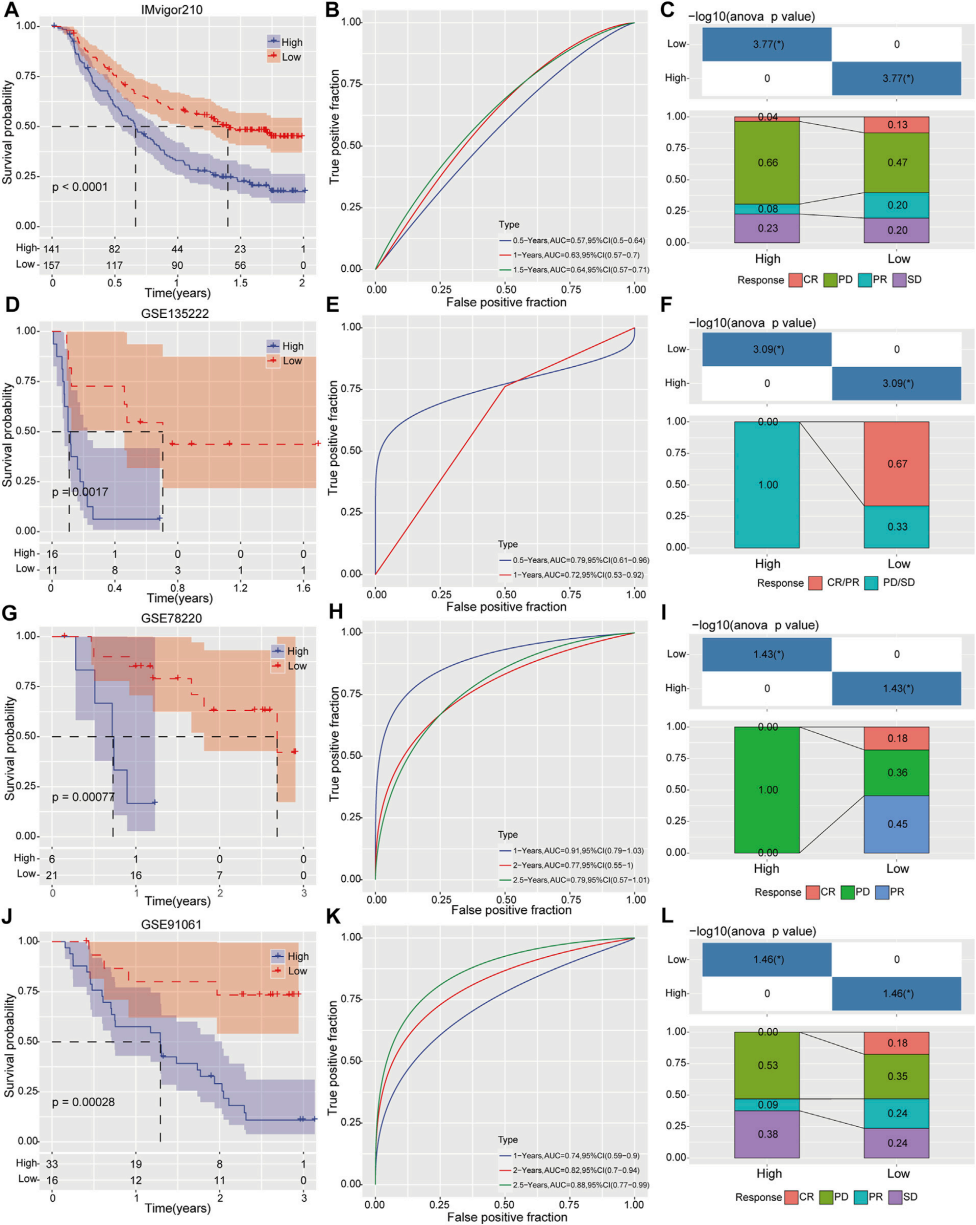
Evaluation prognostic risk model on survival prediction and immunotherapy response. Clinical outcomes and immunotherapy response between high- and low risk groups were determined by Kaplan-Meier analysis, ROC curve and immunotherapy response distribution analysis in IMvigor210 cohort **(A–C)**, GSE135222 cohort **(D–F)**, GSE78220 cohort **(G–I)** and GSE91061 cohort **(J–L)**, respectively.

Four independent clinical cohorts containing gene expression profiles and immunotherapeutic sensitivity profiles were used to estimate our prognostic risk model in drug response. As Kaplan–Meier curves in Figures 7A, D, G, J, patients with high-risk score have inferior survival outcome than those patients with low-risk score. In addition, we further evaluated the predictive accuracy of this risk model. Results displayed that the AUC was 0.64 for 1.5-year OS (95% CI 0.57–0.71) in IMvigor210 cohort (Figure 7B), 0.79 for 0.5-year OS (95%CI 0.61–0.96) in GSE135222 cohort (Figure 7E), 0.91 for 1-year OS (95%CI 0.79–1.03) in GSE78220 cohort (Figure 7H), and 0.88 for 2.5-year OS (95%CI 0.77–0.99) in GSE91061 cohort (Figure 7K), supporting a good predictive performance of this risk model. Intriguingly, the proportion of HCC patients with complete response (CR) and partial response (PR) to immunotherapy in the low-risk category was remarkable elevated than those in the high-risk category as determined in IMvigor210 cohort (Figure 7C). Notably, the high-risk group developed into stable diseases (SD) and progressive diseases (PD) after receiving immunotherapy in GSE135222 cohort (Figure 7F). In GSE78220 cohort, the high-risk category of the cohort only produced PD for immunotherapy, while the probability of low-risk category samples producing CR and PR for immunotherapy was 18% and 45%, respectively (Figure 7I). Similarly, the response rate of the low-risk category to immunotherapy was greatly improved compared with the high-risk category in GSE91061 cohort (Figure 7L). These findings suggests that risk score could significantly distinguish the survival rate at different time points in each immunotherapy cohort.

### Establishing decision tree and nomogram integrated with prognostic risk score and clinicopathological characteristics

**FIGURE 8:**
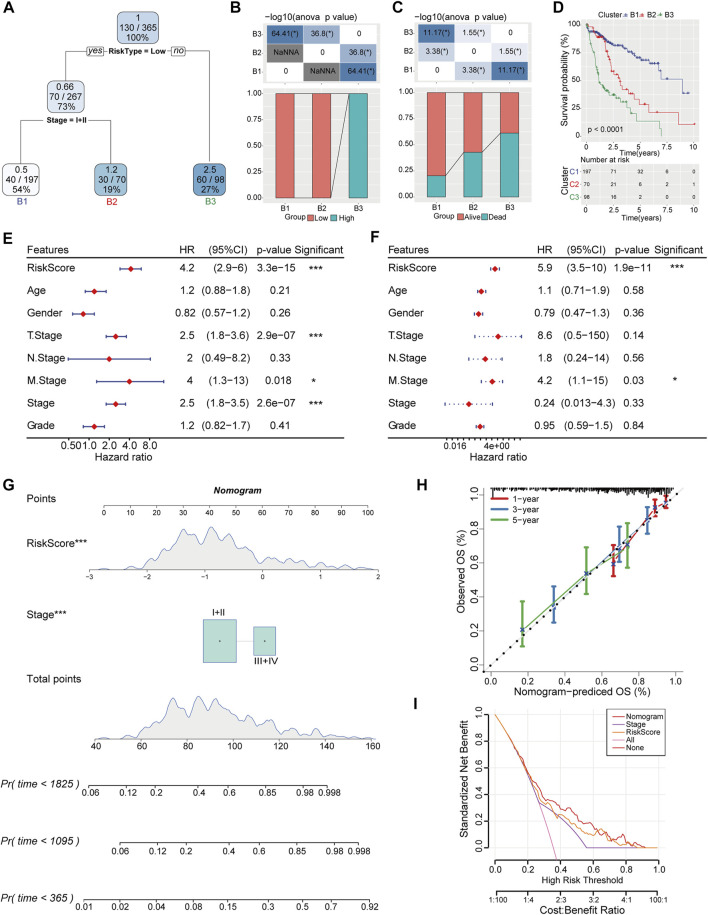
Construction of decision tree and nomogram combined with risk score and clinical pathologic features. **(A)** Decision tree was constructed based on risk score and TNM stage. **(B)** The distribution of different risk categories in each branch. **(C)** Clinical outcomes of patients in two risk groups were estimated in each branch. **(D)** Differences of overall survival rate in three branches were evaluated by Kaplan-Meier analysis. **(E, F)** Univariate and Multivariate Cox regression analysis was conducted to determine the independent risk factor of HCC. **(G)** Nomogram integrated with risk score and TNM stage. **(H)** Calibration diagram for nomogram predicted OS and observed OS in 1, 3, 5-year. **(I)** The reliability of the risk model was evaluated by DCA curves of nomogram.

To further optimize risk stratification system, individuals with full-scale annotations including risk score, gender, age, TNM stage, and clinical grade were enrolled to establish a survival decision tree and three branches were finally generated (Figure 8A). The samples in B1 and B2 branches mainly distributed in low-risk group, while the samples in B3 branch were in high-risk category (Figure 8B). Meanwhile, the clinical outcome of HCC patients classified into branch1 (B1) was better than that in branch3 (B3) (Figures 8C, D). Moreover, risk score and M stage were identified as independent prognostic factor (HR = 5.9, 95% CI = 3.5–10, *p* < 0.001) through Univariate and multivariate analysis (Figures 8E, F). Then, risk score and TNM stage were integrated into nomogram to quantify risk prediction efficiency, and risk score was the main determinant for predicting OS (Figure 8G). The calibration curve demonstrated an accurate goodness of fit between the predicted OS in nomogram and the observed OS (Figure 8H). Meanwhile, the DCA (Decision curve) showed that nomogram and risk score exhibited better prediction ability for OS compared with other factors (Figure 8I).

## Discussion

Nowadays, integrated analysis of epigenetic regulators in HCC has propelled us to recapitulate the vital events in hepatocarcinogenesis. Construction of a genome-scale prognostic risk model for HCC stratification is critical for clinical treatment decision. PRMTs that regulate epigenetic modification, have emerged as key factors in cancer progression, with potential value to be biomarkers for tumor classification, prognosis and drug targeting (Kunadis et al., 2021). Therefore, we conducted the current work to obtain a detailed classification of HCC based on PRMT-related molecules and establish tools for HCC prognosis and therapeutic efficacy evaluation. To the best of our knowledge, this is the first study to use PRMT family-related genes in cancer classification.

To identify the role of PRMTs underlying hepatocarcinogenesis, we stratified HCC patients into three subtypes after analyzing the expression status of PRMTs and screening the PRMT-related genes with potential prognostic value in TCGA-LIHC cohort. The clinical outcomes among these patients were significantly different. Further, we identified 11 PRMT-related prognostic signatures including ECT2, KIF20A, CENPA, TOP2A, CCNB2, PKM, AURKB, CDC20, G6PD, SLC22A1, and ADH4 and constructed a prognostic risk model with high predictive performance. These observations imply a key regulatory role of PRMTs in HCC. Interestingly, several reports have reported that PRMTs can modulate HCC tumorigenesis. For instance, PRMT2 methylates histone H3R8 to accelerate tumorigenesis of HCC (Hu et al., 2020). PRMT3 promotes HCC growth by enhancing arginine methylation of LDHA (Lei et al., 2022). And PRMT4 participates in regulating glucose metabolism in HCC (Zhong et al., 2018). These studies provide strong evidence for PRMTs as promising prognostic biomarker and therapeutic target of HCC.

As a typical inflammation-driven cancer, immune escape is a characteristic that should be highlighted in the occurrence and deterioration of HCC (Fu et al., 2019). In clinical, only a proportion of patients could benefit from immunotherapy, due to high immunotype heterogeneity of HCC (Dodson et al., 2020). Therefore, identifying reliable predictors of immunotherapy response will greatly benefit HCC clinical treatment. Here, we evaluated the immunophenotype and found that the infiltrated immune cells were distinctly different among three HCC subtypes. C3 group, with the worst prognosis among three subtypes, had high degree of adaptive immunity. Thus, we speculated that immune escape might occurred in C3 subgroup. Meanwhile, both the drug sensitivity and immunotherapeutic response were obviously different in two risk categories. Our findings suggested that stratification of HCC might help to understand the immunological characteristics in different subtypes and assistant clinical decision.

Despite great advances for immunotherapy in cancers, the majority of patients relapse or fail to response to immunotherapy due to insufficient immune response against tumors or suppressive tumor microenvironment (TME) (Falcomatà et al., 2023). PRMTs have been elucidated as key factor in tumor immunosurveillance of TME (Dai et al., 2022; Fedoriw et al., 2022). Here, the molecular tool constructed in our study had excellent predictive ability in immunotherapy efficacy and drug sensitivity of HCC. Furthermore, the PRMT-related key genes included in our risk model have been demonstrated involved in tumor immune response regulation. For instance, G6PD was clarified participating in cytotoxic T lymphocytes (CTLs) activation and immune escape in epithelioid mesothelioma (Lu et al., 2022). KIF20A was closely associated with immune infiltration in clear cell renal cell carcinoma (ccRCC) (Ren et al., 2020). CCNB2 and AURKB were negatively correlated with B cells, macrophages, myeloid dendritic cells and CD4^+^T-cell infiltration in lung adenocarcinoma (Xu et al., 2022). CDC20 was confirmed to be involved in the infiltration of cancer-related fibroblasts and myelogenous suppressor cells (Wu et al., 2021). CENPA, TOP2A, and PKM2 were evidenced to regulate immune cell activation in TME (Xu et al., 2021b; Chen et al., 2021; Wang et al., 2022). Notably, ECT2 and ADH4 have been deciphered in promoting M2 macrophages polarization and regulating B cell infiltration in HCC (Xu et al., 2021a; Zhang et al., 2023). Our findings are in line with these studies and supports for detailed mechanistic studies on PRMT-related targets and their associated immune regulation in HCC.

Our study has several limitations. First, the current work was mainly performed by bioinformatic analysis, the predictive results of our risk model might be insufficient. Second, we only confirmed the expression status of PRMTs in HCC TMA cohort and cell lines, further validations with large clinical cohort and rigorous experiments are needed in the following work.

In conclusion, HCC could be divided into three subtypes based on PRMT-related genes. The prognostic risk model constructed in this context provide effective guidance for evaluating HCC patient’s prognosis, drug sensitivity, and immunotherapeutic response. The findings of our systems-level analysis may be desirable for understanding the molecular mechanism underlying HCC, and open new avenues for development of effective treatment strategies.

## Materials and methods

### Expression datasets

The expression profile, clinical information, and single nucleotide variation (SNV) data were downloaded from the TCGA-LIHC project as the training set for this study. GSE14520 and GSE76427 datasets obtained from the Gene Expression Omnibus (GEO) database (https://www.ncbi.nlm.nih.gov/geo/) and HCCDB dataset retrieved from The Integrative Molecular Database of Hepatocellular Carcinoma (HCCDB) database (http://lifeome.net/database/hccdb/home.html) were used as validation sets.

### Screening of PRMT family related genes to conduct consensus clustering

PRMT-related genes were identified using Pearson correlation analysis in TCGA-LIHC dataset and HCCDB18 dataset. The genes that meet the condition of R > 0.7 and *p* < 0.05 were thought to be PRMT related genes. Overlapping PRMT related genes between TCGA-LIHC dataset and HCCDB18 dataset was taken into consensus clustering analysis to classify the molecular subtypes of HCC. “ConsensusclusterPlus” package was used for consensus clustering analysis and the output results included consensus matrix, cumulative distribution function (CDF) and CDF Delta area curves. The rationality of classification was confirmed by principal component analysis (PCA).

### Differential expression analysis between molecular subtypes and construction of prognostic grouping system

The differentially expressed genes (DEGs) between molecular subtypes were analyzed by “LIMMA” package, and the DEGs were overlapped by Venn diagram. The shared DEGs was input to the “survival” package for univariate cox regression analysis. The overfitting genes were excluded by least absolute shrinkage and selection operator (LASSO) penalty regression analysis. Then, multivariate cox regression model was used to reduce data size. Based on the regression coefficient obtained by LASSO regression analysis, a suitable prognostic grouping system: Risk score = Σ [coef (I) * Exp (I)] was formed.

### Mutation analysis

“maftools” package was used to analyze the mutation data retrieved from TCGA. The mutation status in samples was determined and the genes with the lowest mutation frequency >3 were selected. Fisher’s exact test was used to identify the high frequency mutation genes in different subgroups, with *p* < 0.05 as the cutoff value, and the results was visualized as a waterfall map.

### Pathway enrichment analysis

According to gene set enrichment analysis (GSEA) database (Subramanian et al., 2005), single sample GSEA (ssGSEA) was adopted to determine the enrichment score of the pathway in the file.

### Analysis of immune cell infiltration and immune response

Immune cell infiltration was evaluated by ssGSEA, CIBERSORT (Newman et al., 2015) and ESTIMATE (Yoshihara et al., 2013). The immune abundance of each sample was quantified using ssGSEA and the relative proportions of each immune cell type were shown as enrichment scores. Based on the gene expression in TCGA-LIHC cohort, ESTIMATE deduced the proportion of stromal cells and immune cells in the form of stromal score and immune score. Immune response status was evaluated using Tumor Immune Dysfunction and Exclusion (TIDE) (Jiang et al., 2018).

### The prognostic grouping system was verified in immunotherapy cohorts

Four datasets including IMvigor210, GSE91061, GSE78220, and GSE135222 were enrolled for immunotherapy analysis. Prognostic grouping system was applied to calculate risk sore and evaluate HCC patients’ overall survival (OS) rate. receiver operating characteristic (ROC) analysis was employed to examine the area under the curve (AUC) of prognostic risk model.

### Construction of decision tree and nomogram

Classification tree algorithm was employed to construct a decision tree with root nodes, internal leaf nodes and branches based on all the clinical information provided by TCGA and prognostic grouping system. Univariate and multivariate Cox regression analysis were utilized to determine the independent prognostic variables for HCC. Risk score and AJCC TNM stage were fitted to establish a nomogram based on the R package of “rms”, and decision curve analysis (DCA) was carried out by “DCA” package.

### Tissue microarray (TMA) cohort

The TMA cohort including 80 paired HCC tissues and adjacent non-tumor tissues was established in our laboratory. This research was approved by the ethics committee of the First Affiliated Hospital of Zhengzhou University.

### Cell lines and cell culture

Three HCC cell lines including HepG2, Hep3B and Huh7 as well as the normal-type hepatocyte L02 cell line were purchased from the Shanghai Cell Bank of the Chinese Academy of Sciences (Shanghai, China). Cells were cultured in an atmosphere of 5% CO_2_ at 37°C, and maintained in DMEM or RPMI 1640 medium (Gibco) supplemented with 10% fetal bovine serum (Gibco).

### Immunohistochemistry (IHC)

The expression status of PRMTs in tumor and adjacent normal tissues was determined by IHC staining on HCC tissue microarray (TMA) as previously described. Two pathologists counted cells containing brown granules independently. The total score (range from 0 to 15) was calculated by multiplying two scores for the staining intensity (3, strong; 2, moderate; 1, mild; and 0, none) and percentage of positive cells (5, >80%; 4, 80%–61%; 3, 60%–41%; 2, 40%–21%; 1, <20%). Antibodies used in this study are listed in Supplementary Table S1.

### Western blotting

Cells were lysed and total proteins were extracted with RIPA buffer (Solarbio, China). Protein concentration was detected using the BCA protein Quantification Kit (Beyotime, China), and 20 µg protein samples were subjected to SDS–PAGE. Subsequently, proteins were transferred to a PVDF membrane, blocked with 5% non-fat milk, and incubated with primary antibodies at 4°C overnight. After incubating with corresponding secondary antibodies for 1 h at temperature, the membranes were visualized by LumiGLO enhanced chemiluminescent (ECL) or the Odyssey Infrared Imaging System (LI-COR Bioscience, Lincoln, NE). Detailed information on antibodies is provided in Supplementary Table S1.

### Statistical analysis

All statistical analysis in this study was completed by R program. The variables difference between two groups was quantified by Wilcoxon rank-sum test and Student’s *t*-test. Kaplan-Meier analysis was performed using “survminer” package, and the ROC analysis was conducted by the “timeROC” package. Univariate and multivariate cox regression were conducted in the “survival” package. *p* < 0.05 was designated as the statistically significant threshold.

## Data Availability

The datasets presented in this study can be found in online repositories. The names of the repository/repositories and accession number(s) can be found in the article/Supplementary Material.
