# Supplementary material for: Classification molecular subtypes of hepatocellular carcinoma based on PRMT-related genes
Source: Front Pharmacol. 2023 Feb 22;14:1145408. doi: 10.3389/fphar.2023.1145408 (PMC9992644; doi:10.3389/fphar.2023.1145408)
Supplement: Supplementary file 1 [file Table1.DOCX]

| **Antibody** | **IHC** | **Western blot** | **Specificity** | **Company** |
| --- | --- | --- | --- | --- |
| PRMT1 | 1:200 | 1:1000 | Rabbit | Proteintech |
| PRMT2 | 1:200 | 1:1000 | Rabbit | Abclonal |
| PRMT3 | 1:200 | 1:1000 | Rabbit | Abclonal |
| PRMT4 | 1:200 | 1:1000 | Rabbit | Abclonal |
| PRMT5 | 1:200 | 1:1000 | Rabbit | Abclonal |
| PRMT6 | / | 1:800 | Rabbit | Proteintech |
| PRMT7 | 1:200 | 1:1000 | Rabbit | Abclonal |
| PRMT8 | / | 1:800 | Rabbit | Proteintech |
| PRMT9 | 1:100 | 1:1000 | Rabbit | Abclonal |
| GAPDH | / | 1:5000 | Mouse | Proteintech |

**Supplementary table 1**. Information on antibodies used in this study.
