# Supplementary figures and images for: Classification molecular subtypes of hepatocellular carcinoma based on PRMT-related genes
Source: Front Pharmacol. 2023 Feb 22;14:1145408. doi: 10.3389/fphar.2023.1145408 (PMC9992644; doi:10.3389/fphar.2023.1145408)

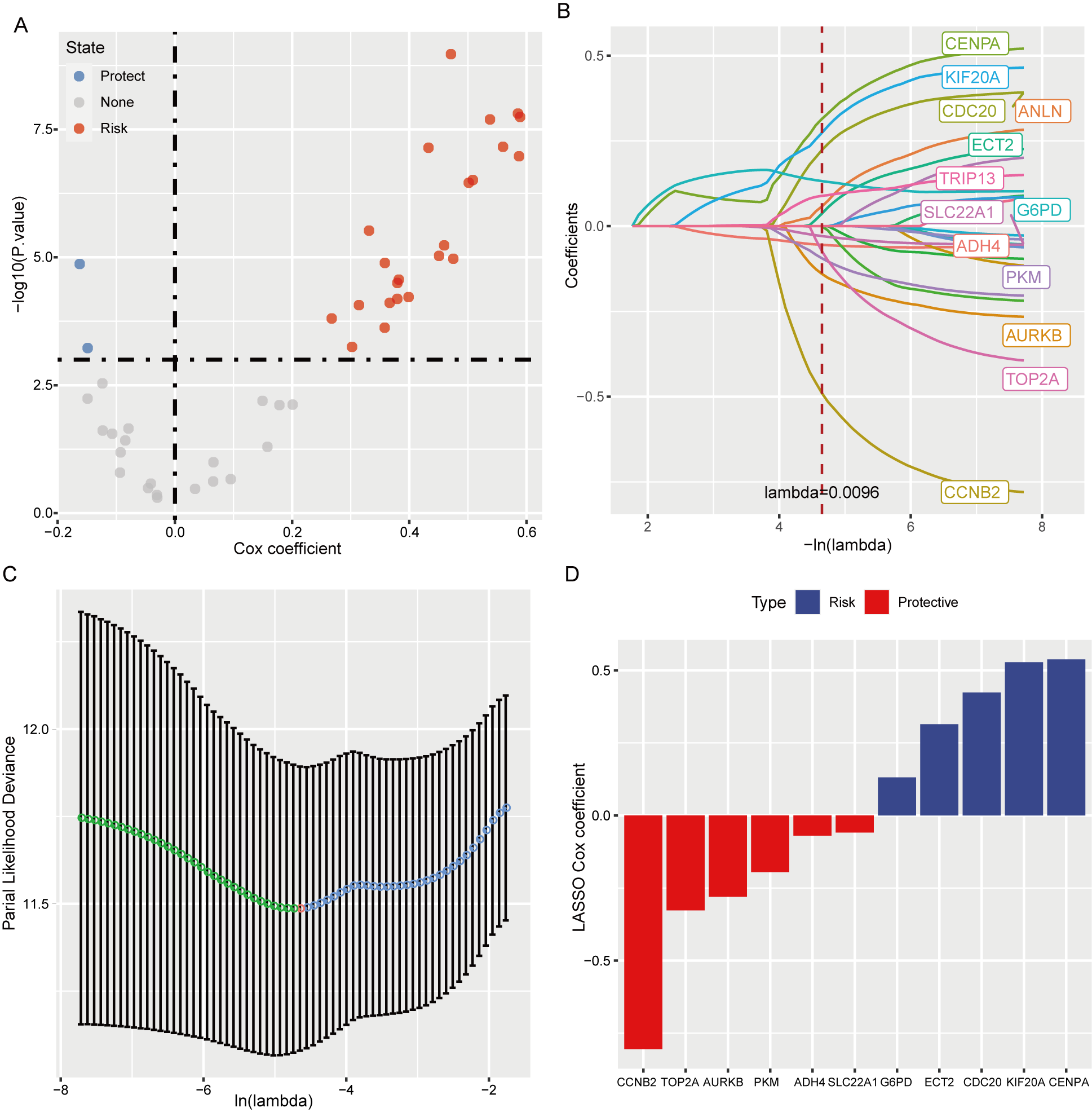

Supplement: Supplementary file 2 [file Image3.TIF]

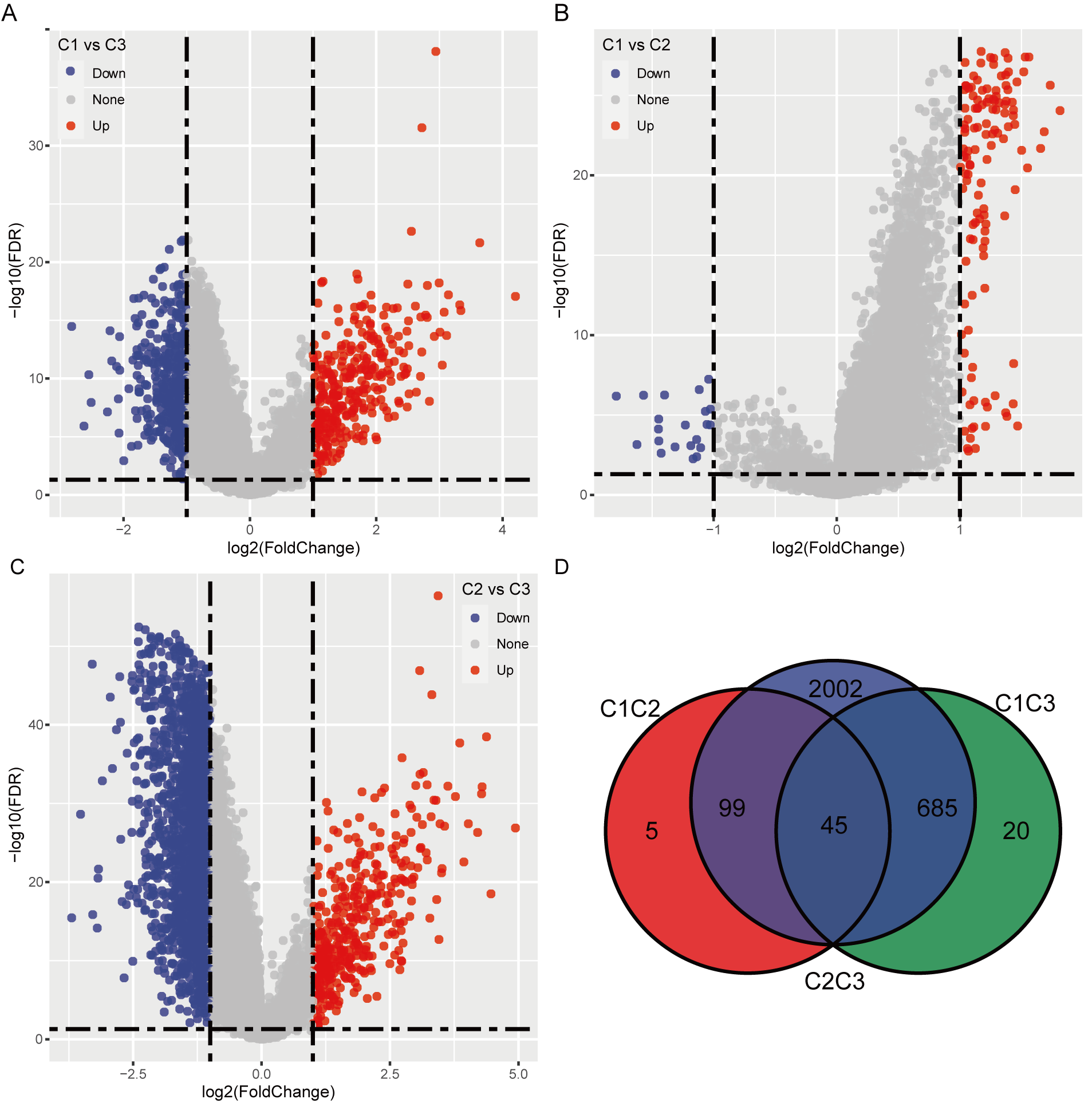

Supplement: Supplementary file 3 [file Image2.TIF]

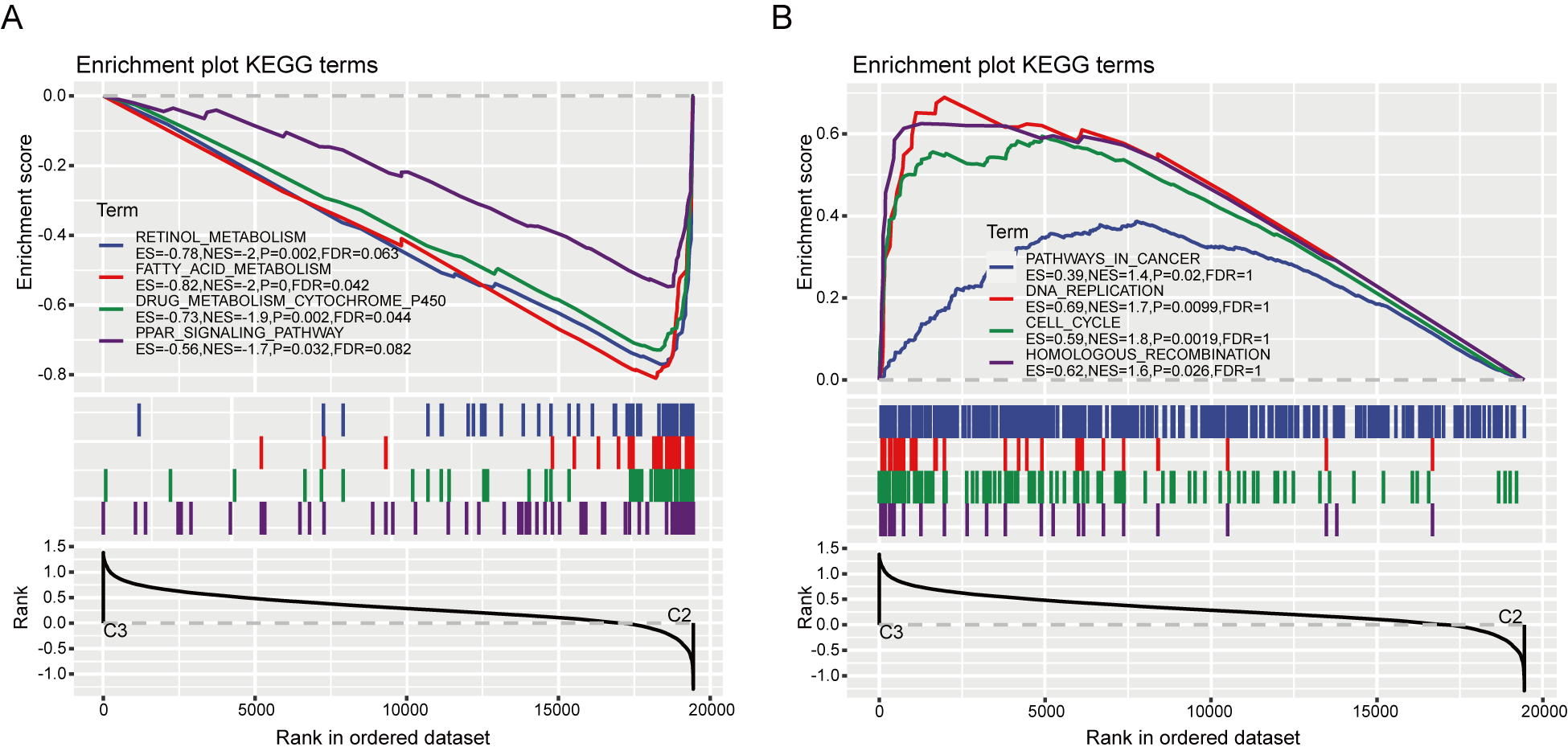

Supplement: Supplementary file 4 [file Image1.TIF]
